# Hallux valgus in preprofessional adolescent dancesport athletes: Prevalence and associated training factors

**DOI:** 10.1002/jfa2.12043

**Published:** 2024-07-30

**Authors:** Zijian Liu, Takumi Okunuki, Hiroki Yabiku, Shuo Chen, Takuma Hoshiba, Toshihiro Maemichi, Yanshu Li, Tsukasa Kumai

**Affiliations:** ^1^ Graduate School of Sport Sciences Waseda University Saitama Japan; ^2^ Research Organization of Science and Technology Ritsumeikan University Kusatsu Japan; ^3^ Research Fellow of Japan Society for the Promotion of Science Tokyo Japan; ^4^ Department of Orthopedic Surgery Graduate School of Medicine University of the Ryukyus Nishihara Japan; ^5^ Faculty of Sport Sciences Waseda University Saitama Japan; ^6^ Graduate School of Human Sciences Waseda University Saitama Japan

**Keywords:** dancesport athlete, hallux valgus, training factor

## Abstract

**Background:**

This study aimed to determine the risk factors of hallux valgus angle among preprofessional adolescent dancesport athletes.

**Methods:**

A total of 275 athletes, (73 males and 202 females) aged between the ages of 11 and 18 years, participated in this study. A cross‐sectional questionnaire was used to survey their demographic characteristics (sex and age), training information (starting age, weekly training time, and athletic career [number of years of training at this specific dancesport school]), and measured their height and weight. The hallux valgus angle was measured based on foot photographs. The chi‐square test was used to compare the difference with prevalence of hallux valgus between male and female athletes. A normal distribution test was performed, and based on the test results, unpaired *t*‐test and multiple logistic regression were conducted to identify training factors for the hallux valgus in this cohort.

**Results:**

Chi‐square test showed higher prevalence of hallux valgus in female elite adolescent dancesport athletes than males. The *t*‐test results did not show any significant differences between the hallux valgus group and non‐hallux valgus groups with start age, athletic career, and weekly training time. Multiple logistic regression analysis with hallux valgus as the dependent variable revealed that the female sex was a strong predictor of a higher prevalence of hallux valgus (odds ratio [OR]: 3.954, 95% confidence interval 95% CI: 2.193–7.131, and *p* < 0.001). Weekly training time was also entered into the multiple logistic regression model (OR: 1.033, 95% CI: 1.001–1.067, and *p* = 0.041).

**Conclusions:**

Our findings revealed that the prevalence of hallux valgus in adolescent dancesport athletes was higher in females than in males. Longer weekly training time was also a risk factor for hallux valgus. Training factors should be considered in preventive programs for elite adolescent dancesport athletes, and special attention should be paid to female athletes.

## 1 INTRODUCTION

Dancesport, a popular worldwide dance competition [1], is divided into three different disciplines: Latin‐American dances (samba, cha‐cha‐cha, rumba, paso doble, and jive), standard dances (waltz, tango, Viennese waltz, slow foxtrot, and quickstep), and 10 dances (five standard and five Latin‐American dances) [2]. In dancesport, male and female athletes wear high heels (males: 3 cm and females: 7 cm) and compete with men and women pairs in terms of performance, techniques, and artistic elements [3].

Hallux abducto valgus, an initially concept introduced by Carl Heuterin in 1871, is a deformity of the first metatarsophalangeal joint [4]. Hallux valgus is a progressive deformity ranging from mild to severe, and its occurrence is influenced by intrinsic and extrinsic factors [5]. Extrinsic factors are footwear (high heels and narrow shoes) [6] and excessive loading [7]. In contrast, intrinsic factors include sex (female > male), genetics [8], age (older > younger) [9], and muscle imbalance between the abductor hallucis and adductor hallucis muscles [10] as well as ligamentous laxity [11]. No association has been found between the occurrence of hallux valgus and any occupation, except dance.

**FIGURE 1:**
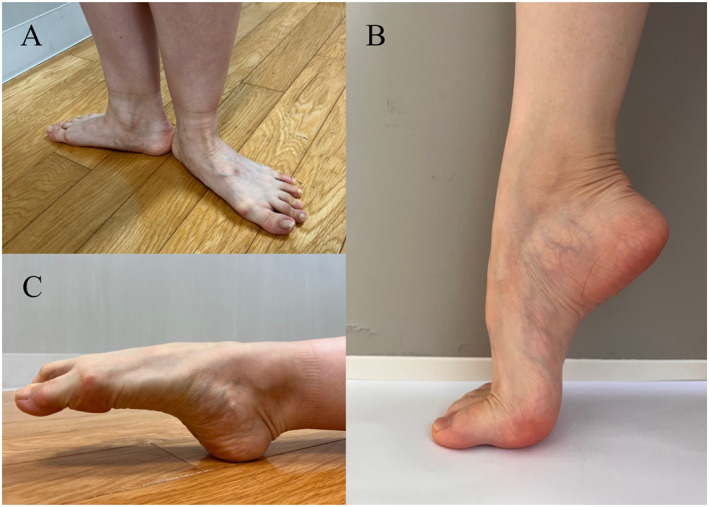
(A) First position. (B) Demi‐pointe. (C) Pointe movement.

A dance technique requires the dancer to make beautiful dances and body movements using the foot. In dancesport, performance based on external rotation of the lower extremities is also required. The representative action of turnout, the external rotation of the lower extremities in dance [12], is the first position (Figure 1A). The first position may lead to midfoot abduction [13] and dropping of the medial arch [14], and dancers are more likely to pronate about the foot/ankle complex in the first position [15]. There is evidence supporting the hypothesis that extreme medial rotation of the first metatarsal may be an important factor in pathogenesis of hallux valgus. Furthermore, extreme medial rotation has also been reported to lead to medial soft tissue failure in the first metatarsophalangeal joint [16, 17]. A previous study reported the hallux valgus occurs because of damage to the first metatarsophalangeal joint and due to changes in the midfoot and medial cuneiform bone caused by pronation of the first metatarsal bone [18]. Dancers frequently use demi‐pointe and en‐pointe (only in ballet, requiring pointe shoes) movements (Figure 1B). However, two previous studies reported the foot while en‐pointe or demi‐pointe may result in microtrauma of the metatarsophalangeal joint capsule and predispose the dancer to hallux valgus [19, 20]. In addition, to achieve the required aesthetics, the “pointe” technique is frequently used in dancing (Figure 1C). The “pointe” technique must perform motions of extreme ranges, particularly in plantar flexion, where 90°–100° is considered optimal [21]. To effectively perform the “pointe” technique, it is necessary to achieve the maximum plantar flexion of the foot joint with contributions from the ankle (more than 80%) as well as the Chopart's (10.8%) and Lisfranc (6.7%) joints [22]. Furthermore, to complete the technique, all the toes, particularly the first metatarsophalangeal joint, should be maximally flexed [21]. A previous study reported that adductor hallucis muscle contributes more than the abductor hallucis muscle during flexion of the first metatarsophalangeal joint [23]. Therefore, the “pointe” technique may facilitate the muscle imbalance between the adductor hallucis and abductor hallucis muscles, leading to hallux valgus. In 1928, Morton was the first to introduce the concept that hypermobility may contribute to foot deformity [24]. Two studies reported the prevalence of joint hypermobility among dancers, which is higher than that in the general population [25, 26]. Another study reported that hallux valgus severity is correlated with increased mobility of the first tarsometatarsal joint [11]. Hypermobility of the first ray is a probable primary cause of hallux valgus [27]. In summary, the physical traits and characteristics of dance may explain the high prevalence of hallux valgus among dancers.

Dancesport athletes wear high heels during training and competitions, which is a risk factor for hallux valgus. Wearing high heels increases the plantar pressure in the head of regions of the first and second metatarsal bones head regions more than that observed with flat and running shoes [28]. High heels also cause excessive overload on the forefoot and increase the pressure on the second to third metatarsal bones, thereby lowering the transverse arch [29]. Furthermore, wearing high‐heel and narrow‐toe box shoes overloads the great toe from the medial side, resulting in first metatarsophalangeal joint adduction and increasing the valgus moment [30]. The risk of hallux valgus is higher in women who habitually wear high heels than in those who habitually wear flat shoes [31]. Therefore, wearing high heels is also a risk factor for hallux valgus in dancesport athletes and may be the cause of the higher prevalence of hallux valgus than that observed in other types of dances.

In the general population, hallux valgus influences patients' quality of life [32], balance [33, 34], and physical performance [35]. Findings from previous studies have consistently demonstrated that patients with hallux valgus exhibit a decreased loading of the hallux during the gait cycle, potentially attributed to a functional impairment in the first metatarsophalangeal joint [36, 37, 38]. Similar findings were observed in dancers, as a previous study demonstrated the detrimental impact of hallux valgus on dancers' quality of life [39]. Furthermore, an inverse relationship was identified between the degree of hallux abduction and the load‐bearing capacity during demi‐pointe movement [40]. In another study, hallux valgus was shown to lead to an enlarged retroversion of the pelvis during a plié [41]. Hence, it is imperative to explore the incidence and prevalence of hallux valgus among dancers. However, there are currently no studies reporting the prevalence of hallux valgus in dancesport athletes.

This study aimed to determine the prevalence of hallux valgus and risk factors for hallux valgus in elite adolescent dancesport athletes. We hypothesized that high prevalence of hallux valgus among adolescent preprofessional dancesport athletes is associated with sex and training factors.

## 2 MATERIALS AND METHODS

### 2.1 Participants

This study was conducted at a specialty dancesport school in China. We enrolled 366 adolescent dancesport athletes who underwent the same training program at this school. Participants were excluded based on the following criteria: (1) those who wore high heels even when not dancing (≧2 days weekly), (2) those who had a history of lower extremity injury or disorder such as a fracture in the last 12 months, and (3) those who had injuries and symptoms before entering the specialty dancesport school.

**FIGURE 2:**
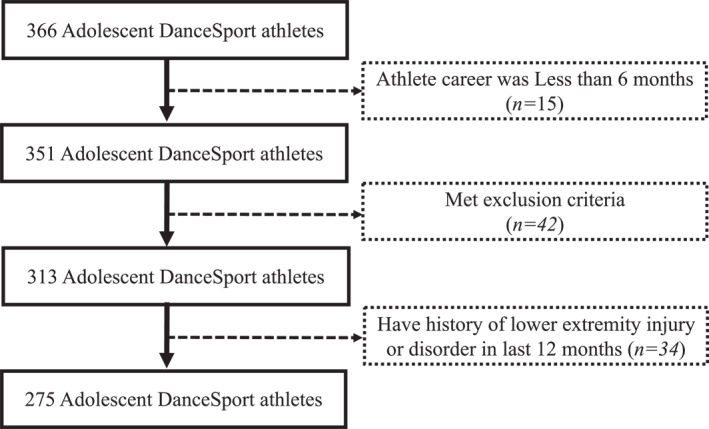
Participant flowchart for study inclusion.

The volunteers were interviewed before the survey and those who had a history of surgery or were advised by a doctor to temporarily stop practice within the last 12 months were excluded. Finally, 275 athletes were selected for this cross‐sectional study (Figure 2). The survey was conducted after the athletes and their parents provided consent. Ethical approval for this study was granted by the Waseda University Human Research Ethics Committee (No. 2019‐343).

### 2.2 Questionnaire

A simple self‐administered questionnaire was used for the survey. Two physical therapists explained the questions to the athletes to ensure that they provided accurate answers. The questionnaire included questions regarding demographic data (sex and age) and training information such as starting age (the age at which athletes began training at this specific dancesport school), weekly practice time (hours/week, including time in lessons and individual practice), and athletic career (number of years of training at this specific dancesport school).

### 2.3 Anthropometric measurement

The height and weight of all participants were measured and their body mass index (BMI) was subsequently calculated. For height measurement, the participants stood on the Seca 213 portable stadiometer (Seca GmbH and Co. KG) with standard anatomical posture ensuring that the buttocks and the back of their heads were in contact with the machine. The digital weighing scale (OMRON, HN‐289) was used to measure weight with a similar position. BMI was calculated as body weight (kg) divided by height (m) squared.

### 2.4 Measurement of hallux valgus angle

For the measurement of hallux valgus angle, we used foot photographs. The participants stood upright in a normal standing position with their feet shoulder‐width apart, hands on the anterior superior iliac spine, and eyes looking forward. A measurement camera (Canon, SX430is) was set in position at an inclined angle of 15° relative to the vertical line passing through the tip of the second toe, and a still picture was taken [33].

**FIGURE 3:**
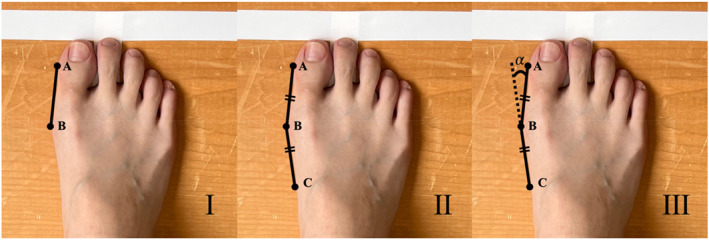
The method of hallux valgus angle measurement. (A) The medial edge of the hallux. (B) The medial edge of the first metatarsal bone head. (C) The medial edge of the first metatarsal bone defined as the length of AB = BC. The angle formed by AB and the extended line of BC.

The hallux valgus angle was measured by an investigator with 5 years of experience in hallux valgus angle research using ImageJ (Ver. 1.53k). First, a tangent line (AB) was drawn from the medial edge of the hallux (A) to the medial edge of the head of the first metatarsal bone (B) (Figure 3I). Subsequently, a line was marked along the medial edge of the foot as points A, B, and C such that lines AB and BC were equidistant from contact point B along the medial edge of the first metatarsal (Figure 3II). The angle (*α*) between lines AB and BC (extended line) was the hallux valgus angle (Figure 3III) [33].

Following a report by a previous study that the photographic hallux valgus angle degree was lower than the radiographic hallux valgus degree (−5.3°; [95% confidence interval [CI]: −4.3–6.2]) [42], we set the cut‐off for hallux valgus at an angle of 20.0° (radiographic hallux valgus degree) and classified participants into the hallux valgus group (photographic hallux valgus angle of 1 or both feet ≥14.7°) and the non‐hallux valgus group (photographic hallux valgus angle of both feet <14.7°) [33].

For the measurement reliability of the hallux valgus angle, we randomly selected 100 participants to estimate the intra‐rater reliability of the hallux valgus angle measured with graphs using intraclass correlation coefficient (ICC1, 3) estimates [43]. Thresholds of reliability for ICC values were defined as poor (<0.50), moderate (0.50–0.75), good (0.75–0.90), and excellent (>0.90) [43]. In this study, the ICC (1, 3) value was 0.941, indicating excellent hallux valgus angle measurement.

### 2.5 Statistical analysis

Statistical analysis was performed using the SPSS version 29.0 (IBM Corp.). The chi‐square test was employed to assess the difference in prevalence between male and female athletes. The Shapiro–Wilk test was used to assess the normality of the training information and hallux valgus angle. To test the differences between the male and female athletes, an unpaired *t*‐test was performed if the data were normally distributed. Otherwise, the Mann–Whitney *U* test was used to test the data that were not normally distributed. After conducting the univariate analysis, we performed multiple logistic regression and calculated the 95% CI for multiple logistic regression in this study. For all tests, the significance level was set at *p* < 0.05.

## 3 RESULTS

The characteristics of 275 dancesport athletes (73 males and 202 females), including height, weight, BMI, starting age, athletic career, and weekly practice time are provided in Table 1. The overall prevalence of hallux valgus was 54.9% (*n* = 151) among all elite adolescent dancesport athletes. Specifically, 32.9% (*n* = 24) among male and 62.9% (*n* = 127) among female athletes.

**TABLE 1:** Characteristics of 275 elite adolescent dancesport athletes.

Subject	Male	Female
Age	16.2 ± 1.4	15.5 ± 1.5
Height (cm)	175.1 ± 5.1	163.3 ± 5.5
Weight (kg)	60.0 ± 8.3	49.4 ± 5.7
Body mass index (BMI)	27.4 ± 7.5	23.8 ± 6.1
Start age (years)	13.4 ± 1.5	13.2 ± 1.4
Athletic career (years)	2.8 ± 1.7	2.3 ± 1.4
Weekly training time (hours/week)	34.5 ± 8.4	31.1 ± 7.9

A significant difference was observed between the sexes using the chi‐square test (*p* < 0.001). Although a tendency for longer training times was observed in the hallux valgus group, the unpaired *t*‐test showed no significant differences in starting age, weekly training time, and athletic career between the study groups.

Multiple logistic regression analysis revealed that female sex was a strong predictor of hallux valgus with an odds ratio (OR) of 3.954 (95% CI: 2.193–7.131 and *p* < 0.001). Longer weekly training time (OR: 1.033, 95% CI: 1.001–1.067, and *p* = 0.041) was also included in the multiple logistic regression model (Table 2).

**TABLE 2:** Multivariable logistic regression analysis of HVAp.

	Hallux valgus (14.7° classification)				
	*B*	OR	CI_95_		*p*‐value
Weekly training time	0.033	1.033	1.001	1.067	0.041
Sex	1.375	3.954	2.193	7.131	<0.001
Constant	−3.236	0.039	/		<0.001

## 4 DISCUSSION

Based on a cross‐sectional survey of elite adolescent dancesport athletes, we investigated the association between sex, training information, and hallux valgus occurrence. Our results revealed that sex (female) and longer weekly training time were significantly correlated with hallux valgus occurrence.

According to a previous meta‐analysis, which evaluated the pooled prevalence estimates, the overall prevalence of hallux valgus was 7.8% in juveniles (males: 5.7% and females: 15.0%), 23.0% in adults (males: 8.5% and females: 26.3%), and 35.7% in older adults (age >65 years, males: 16.0% and females: 36.0%) [44]. However, two studies on younger ballet dancers aged 8–16 years revealed that the prevalence of hallux valgus was 40% (*n* = 1336) [45] and 56.4% (*n* = 55) [39]. Although one study reported no relationship between dancing and hallux valgus prevalence [46], most studies have reported that the prevalence is higher in dancers than in the general population [45, 47, 48]. Based on the result of this study, elite adolescent dancesport athletes appear to have a higher prevalence of hallux valgus than the general population and younger ballet dancers. Dancesport athletes wear high heels during training and competitions. As a result, high heels may contribute to the higher prevalence of hallux valgus compared to other types of dances.

Previous literature revealed a strong association between sex (female) and hallux valgus development [5, 7, 44]. Previous studies have also reported that the general female population had a higher ratio of hallux valgus than their male counterpart [49, 50]. One study reported an anatomical factor that may lead to this increased incidence in females. Specifically, female patients had a more rounded and smaller metatarsal head articular surface than male patients, thereby providing a less stable joint [51]. Notably, a rounder metatarsal head is associated with an increased incidence of hallux valgus deformity. Additionally, female populations also tend to have a more adducted first metatarsal [52], possibly due to the differences in the tarsometatarsal articulation between males and females [53]. The abductor hallucis muscle attaches to the side of the first metatarsal head and inserts on the proximal phalanx, prevoiding abduction and flexion function with the first metatarsal joint [54]. This anatomical factor has a significant relationship with hallux valgus [10]. Kurihara et al. reported a difference between sexes in relation to toe grip and push strength (male > female) [55]. The abductor hallucis muscle abducts and assists in the flexion of the metatarsophalangeal joint of the great toe [56]. Lower muscle strength may contribute to a higher prevalence of hallux valgus in female populations. In this study, we also noted similar results suggesting that female athletes had a higher prevalence of hallux valgus than male athletes.

A history of leg injury has been reported among elite adolescent players with different weekly sports volumes [57], and the injured younger athletes spent more hours per week in organized sports [58]. The risk for injury increases with the number of years spent in dancesport training [59], and the highest perceived cause of injury in dancesport is overtraining [60]. However, almost all the studies have focused on injury rates without addressing specific problems. In this study, we found that the training factor was a risk factor for hallux valgus in elite adolescent dancesport athletes. However, we could not identify a correlation between the hallux valgus and starting age in dancesport. Although a difference in starting age was noted between the noninjury and injury groups in a previous study [59]. A previous study reported the hallux valgus angle increases in children younger than 10 years at 1.5° per year but did not report any significant increase in older children [61]. This demonstrates that the development of adolescent hallux valgus originates at a young age with the increased hallux valgus angle increases afterward [62]. In this study, the average starting age of participants was 13.3 ± 1.4, and may be due to the starting age being over 10 years of age, the start age was not a significant factor in the multivariate logistic regression model.

This study had some limitations. We identified that the hallux valgus angle in the elite adolescent dancesport athletes was influenced by other risk factors, and statistical analysis confirmed that sex and weekly training time were also factors that contributed to hallux valgus occurrence. However, there are two clinical diagnostic standards for hallux valgus (15° and 20°), which may account for the differences in findings across studies. Future research should include adult participants to clarify the influence of starting age on hallux valgus occurrence in elite adolescent dancesport athletes. The results of the multiple logistic regression in this study showed a small effect size. Other risk factors for hallux valgus include footwear [6], genetics [8], and joint hypermobility [25]. However, these risk factors were not surveyed in this study. Future studies should consider factors such as footwear and genetics.

## 5 CONCLUSION

This study identified a high prevalence of hallux valgus among a cohort of preprofessional adolescent dancesport athletes. Female adolescent dancesport athletes had higher hallux valgus prevalence than male athletes and longer weekly training time was a risk factor for hallux valgus. Future research should focus on hallux valgus in elite adolescent dancesport athletes, particularly those with a longer weekly training time. Moreover, special attention should be paid to female athletes.

## AUTHOR CONTRIBUTIONS

**Zijian Liu**: Conceptualization; investigation; formal analysis; methodology; project administration; writing—original draft; writing—review and editing. **Takumi Okunuki**: Formal analysis; investigation; writing—review and editing. **Hiroki Yabiku**: Formal analysis; investigation. **Shuo Chen**: Investigation. **Takuma Hoshiba**: Methodology; investigation. **Toshihiro Maemichi**: Methodology; formal analysis; writing—review and editing. **Yanshu Li**: Writing—original draft; writing—review and editing. **Tsukasa Kumai**: Methodology; investigation; formal analysis; writing—review and editing.

## CONFLICT OF INTEREST STATEMENT

The authors declare no conflicts of interest.

## ETHICS STATEMENT

Ethical approval for this study was granted by the Waseda University Human Research Ethics Committee (No. 2019‐343).

## Data Availability

The datasets generated and/or analysed during the current study are not publicly available, but are available from the corresponding author on reasonable request.
